# AAOCA and ALCAPA Are Distinct Congenital Coronary Anomalies With Different Pathophysiology and Management

**DOI:** 10.31083/RCM49595

**Published:** 2026-08-20

**Authors:** Zewen Chen, Xiaobing Liu, Shusheng Wen, Yong Zhang

**Affiliations:** ^1^Department of Cardiac Surgery, Guangdong Cardiovascular Institute, Guangdong Provincial People’s Hospital, Guangdong Academy of Medical Sciences, Southern Medical University, 510080 Guangzhou, Guangdong, China; ^2^Guangdong Provincial Key Laboratory of South China Structural Heart Disease, 510080 Guangzhou, Guangdong, China

Dear Editor,

In a recent issue of the Reviews in Cardiovascular Medicine, Zhang and colleagues [1] reported their single-center experience with the surgical management of anomalous aortic origin of a coronary artery (AAOCA). In this large case series, patients with anomalous left coronary artery from the pulmonary artery (ALCAPA) were analyzed together with those with right coronary artery arising from the left sinus (R-AAOCA) and left coronary artery arising from the right sinus (L-AAOCA), collectively designated as an “AAOCA cohort”. Based on this grouping, the authors described the clinical presentation, surgical strategies, and outcomes of this population and concluded that ALCAPA accounted for a substantial proportion of AAOCA cases, further emphasizing the benefits of surgical intervention, including in asymptomatic patients. While this work provides valuable surgical experience and outcome data, the inclusion of ALCAPA within the AAOCA cohort raises important concerns regarding disease classification and interpretation of results. Specifically, this grouping is inconsistent with established coronary anomalies (CA) nomenclature and may obscure fundamental differences in anatomy, pathophysiology, indications for intervention, and surgical techniques between AAOCA and ALCAPA. Clarification of these distinctions is essential to avoid potential misunderstanding of both entities and to ensure appropriate clinical decision-making.

CA represents a broad spectrum of congenital disorders with highly variable anatomy and pathophysiology [2]. In 2000, the Congenital Heart Surgery Nomenclature and Database Project defined “CA” as the first hierarchical level, with “CA, Anomalous pulmonary origins of the coronaries” and “CA, Anomalous aortic origins of the coronaries (AAOC)” as parallel second-level entities [3]. Thus, AAOCA and ALCAPA occupy the same hierarchical level rather than a parent–child relationship. Similarly, Angelini P proposed a CA classification integrating all possible coronary anatomic variations in 2007 [2]. In this CA classification, AAOCA belongs to “Anomalous location of coronary ostium at improper sinus”, while ALCAPA belongs to “Anomalous location of coronary ostium outside normal ‘coronary’ aortic sinuses”. This classification places AAOCA and ALCAPA into distinct categories based on coronary ostial location. In a 2023 review in the Journal of the American College of Cardiology (JACC) on AAOCA management, AAOCA was also divided into R-AAOCA and L-AAOCA without including ALCAPA as a subgroup [4]. In addition, a more recent study published in *Circulation* in 2025 on the clinical significance and outcome predictors of AAOCA indicates that ALCAPA is not considered a subgroup of AAOCA [5]. Hence, recent studies generally distinguish AAOCA from ALCAPA.

The pathophysiological mechanisms underlying myocardial ischemia in AAOCA and ALCAPA are fundamentally different. AAOCA refers specifically to coronary arteries arising anomalously from the aorta and includes R-AAOCA and L-AAOCA. Characteristic anatomic features include an acute take-off angle, high take-off, slit-like or elliptical ostium, and anomalous coronary courses such as interarterial, intramural, retroaortic, subpulmonic, or prepulmonic pathways [5]. These anatomic configurations predispose patients to both fixed and dynamic coronary obstruction. Fixed obstruction results primarily from ostial abnormalities and proximal luminal narrowing, whereas dynamic obstruction is related to intramural coronary segments within the aortic wall, myocardial compression, and arterial spasm [4,6]. These mechanisms can lead to episodic myocardial ischemia and malignant ventricular arrhythmias, thereby predisposing patients to sudden cardiac death (SCD), although the presence and severity of ischemia may be variable and inconsistent among individuals [4,6,7]. In contrast, ALCAPA represents an anomalous origin of the left coronary artery from the pulmonary artery and is traditionally classified into infant and adult types [8]. In the infant form, lack of well-developed coronary collaterals, together with the postnatal decline in pulmonary arterial pressure and oxygen content and closure of the ductus arteriosus, results in persistently low coronary perfusion pressure and coronary steal, leading to severe myocardial ischemia and high mortality without surgical correction [9,10,11,12]. In the adult form, which accounts for fewer than 15% of cases, survival into adulthood is facilitated by a dominant right coronary artery and extensive collateralization; however, chronic subclinical ischemia persists, and sudden cardiac death has been reported at a mean age of approximately 35 years [8]. Although myocardial ischemia represents a shared clinical endpoint in both conditions, the underlying mechanisms are distinct. Consequently, clinical presentation and preoperative measures of ejection fraction and left ventricular end-diastolic diameter should not be directly compared between AAOCA and ALCAPA, as pooling these entities may obscure disease-specific pathophysiology and lead to misinterpretation of clinical data.

Indications for intervention differ substantially between AAOCA and ALCAPA and should be considered within distinct clinical and guideline-based frameworks. For AAOCA, indications for surgical intervention are relatively nuanced and depend on coronary anatomy, symptom status, and evidence of myocardial ischemia [4]. The American Heart Association (AHA)/American College of Cardiology (ACC) guidelines provide the strongest recommendation for surgical correction in patients with L-AAOCA, as well as in patients with R-AAOCA who present with clinical symptoms or demonstrable ischemia attributable to the anomalous coronary artery [13]. In contrast, the European Society of Cardiology (ESC) guidelines emphasize high-risk anatomical features in combination with typical anginal symptoms as key criteria for intervention [10]. Notably, most reported cases of SCD associated with AAOCA occur in patients younger than 30 years of age [14]. Consequently, many asymptomatic patients, particularly middle-aged and older adults, may be managed conservatively with careful surveillance and a watchful waiting strategy [4,15]. In pediatric patients with AAOCA, surgical timing is also individualized; a recent study from China reported a median age at surgical intervention of approximately 9 years [16]. In contrast, ALCAPA represents a fundamentally different clinical entity with a distinct and more uniform indication for surgery. Diagnosis of ALCAPA itself constitutes a definitive indication for surgical repair, as most untreated infants die within the first year of life [13,17]. Surgical correction is the only accepted therapy to ensure survival and is therefore typically performed in early infancy [18]. Consistent with the “first-year rule”, the median age at surgical repair in our previous study was approximately 9 months [19]. For the small minority of patients with ALCAPA who survive into adulthood, optimal management remains controversial, with some authors advocating surgical repair and others not considering it the preferred option in all cases [9,20].

Surgical strategies also differ substantially between these two entities. In AAOCA, operative techniques include unroofing, coronary reimplantation, and neo-ostium creation, with the choice of procedure tailored to individual coronary anatomy and the specific features of the anomaly [7]. Although a report from Stanford University School of Medicine identified coronary reimplantation as their preferred approach [21], no single technique is universally applicable, and the surgical strategy should remain anatomy driven. In contrast, the most commonly employed surgical strategy for ALCAPA is coronary reimplantation [22]; however, this procedure entails translocation of the coronary artery from the pulmonary artery to the aorta, which is fundamentally different from AAOCA repair, in which the coronary artery is transected at its anomalous (“false”) aortic origin and reimplanted into the appropriate aortic sinus [7].

In summary, although Zhang and colleagues [1] present valuable clinical data, classifying ALCAPA as a subset of AAOCA is inconsistent with established nomenclature and obscures fundamental differences in anatomy, pathophysiology, indications for intervention, and surgical techniques. AAOCA and ALCAPA should therefore be regarded as distinct coronary anomalies and analyzed separately. Future investigations may more productively focus on unresolved clinical gray zones, such as late-presenting or asymptomatic patients, where optimal management strategies remain debated.
